# Poster Session II - A236 USE OF ASSISTED REPRODUCTIVE TECHNOLOGIES (ART) IN PATIENTS WITH INFLAMMATORY BOWEL DISEASE (IBD): A RETROSPECTIVE STUDY

**DOI:** 10.1093/jcag/gwaf042.236

**Published:** 2026-02-13

**Authors:** H Li, V Srikanth, K O’Connor, L Tang, S Srikanth, J M Lee, V W Huang

**Affiliations:** Division of Gastroenterology, Mount Sinai Hospital, Toronto, ON, Canada; Division of Gastroenterology, Mount Sinai Hospital, Toronto, ON, Canada; Division of Gastroenterology, Mount Sinai Hospital, Toronto, ON, Canada; Division of Gastroenterology, Mount Sinai Hospital, Toronto, ON, Canada; Division of Gastroenterology, Mount Sinai Hospital, Toronto, ON, Canada; Division of Gastroenterology, Mount Sinai Hospital, Toronto, ON, Canada; Division of Gastroenterology, Mount Sinai Hospital, Toronto, ON, Canada

## Abstract

**Background:**

Women with active IBD have an increased risk of infertility and may benefit from Assisted Reproductive Technologies (ART). Pregnancy and live birth rates have been shown to be similar in women with and without IBD undergoing ART. However, guidelines regarding ART referral criteria and overall efficacy of its use in this population are lacking. ART may have decreased efficacy in patients with a history of IBD-related pelvic surgery and the effect of hormonal changes secondary to ART on IBD flares remains to be elucidated.

**Aims:**

This retrospective chart review study at a tertiary centre aimed to explore the use of ART in the preconception period for patients with IBD. Prevalence of ART use and pregnancy outcomes were analyzed.

**Methods:**

All adult patients referred to the Preconception and Pregnancy in IBD Program in 2023 were included. Patients were excluded if they did not have a formal diagnosis of IBD or were seen for non-pregnancy related concerns. For patients with multiple attempted or successful pregnancies, each unique attempt or successful pregnancy was recorded as a separate journey.

**Results:**

A total of 149 unique journeys were identified (Figure 1). Of these, 35 (23.5%) remained preconception at time of analysis. Of those preconception, 16 (45.7%) underwent fertility assessment or utilized ART during their current pregnancy attempt/journey.

Overall, of all 114 conceptions, 15 (13.2%) resulted from ART use and 99 from spontaneous conception (86.8%). Of the 15 ART-assisted conceptions, 13 (86.7%) were via in vitro fertilization (IVF), 1 (6.7%) was via frozen embryo transfer (FET) IVF, and 1 (6.7%) was via ovarian stimulation. Mean age was higher for ART-assisted conceptions compared to spontaneous conceptions (35 vs 32, p = 0.006). There were no statistically significant differences between these two groups with regards to IBD diagnosis (p = 0.931) and biologic use (p = 0.566).

Pregnancy outcomes were available for 97 (85.1%) of the total conceptions, encompassing 14 ART-assisted conceptions and 83 spontaneous conceptions. The remaining 17 (14.9%) had not yet delivered at time of analysis. There was no statistically significant difference between live births (100.0% vs 96.4%, p = 1.00) but fewer ART-assisted conceptions resulted in full-term births (78.6% vs 96.2%, p = 0.042).

**Conclusions:**

Live birth rates were similar in patients with ART-assisted versus spontaneous conceptions but full-term births were lower in the former. The majority of patients undergoing ART conceived via IVF. Patients undergoing ART may be older and represent a more complex population with greater treatment/support needs. Further research with larger sample sizes is required to better evaluate the efficacy of ART in patients with IBD.

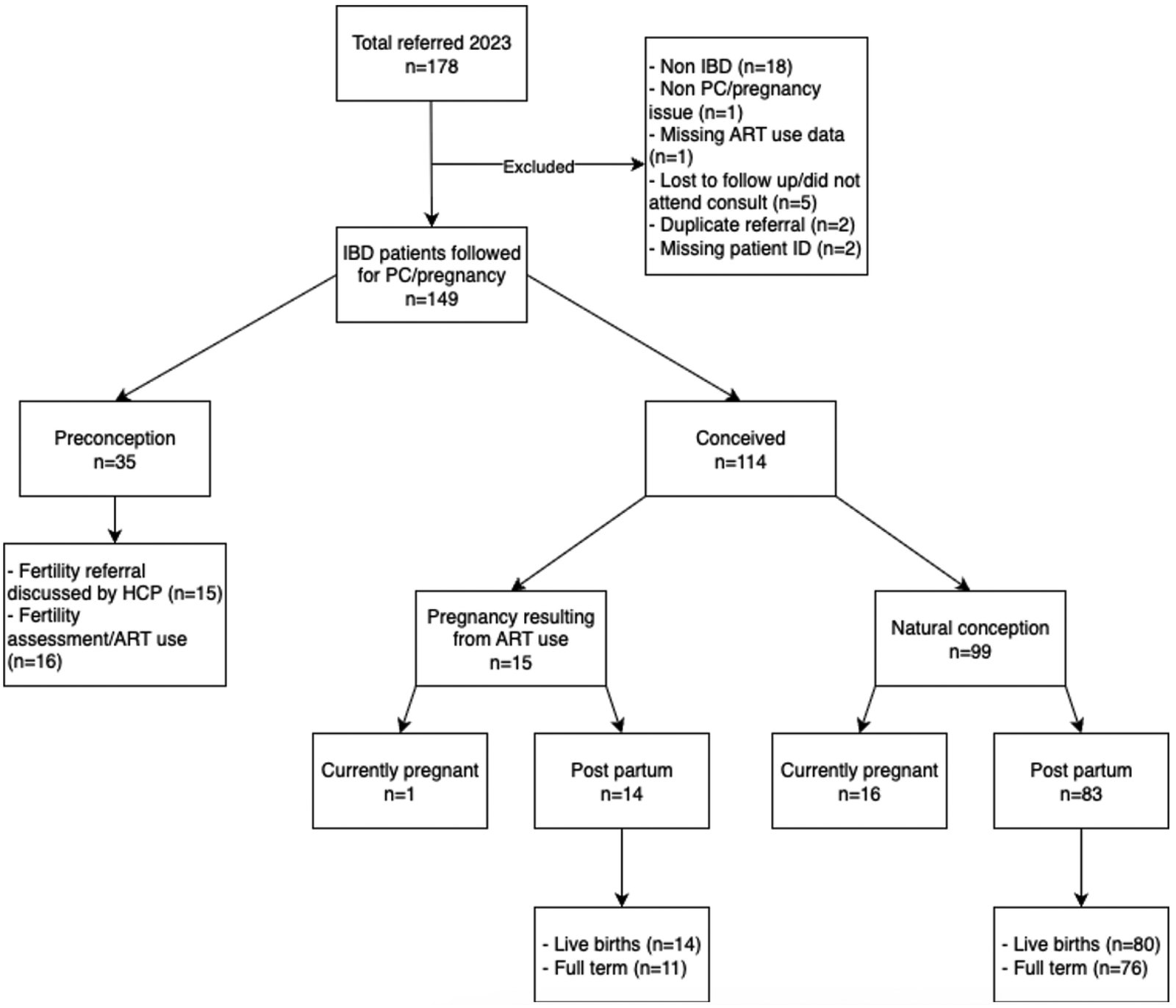

**Funding Agencies:**

None

